# Identification of a Common Lupus Disease-Associated microRNA Expression Pattern in Three Different Murine Models of Lupus

**DOI:** 10.1371/journal.pone.0014302

**Published:** 2010-12-10

**Authors:** Rujuan Dai, Yan Zhang, Deena Khan, Bettina Heid, David Caudell, Oswald Crasta, S. Ansar Ahmed

**Affiliations:** 1 Center for Molecular Medicine and Infectious Diseases (CMMID), Department of Biomedical Sciences and Pathology, Virginia-Maryland Regional College of Veterinary Medicine, Virginia Polytechnic Institute and State University, Blacksburg, Virginia, United States of America; 2 Virginia Bioinformatics Institute, Virginia Polytechnic Institute and State University, Blacksburg, Virginia, United States of America; University of California San Francisco, United States of America

## Abstract

**Background:**

Recent reports have shown that microRNAs (miRNAs) regulate vital immunological processes and have emerged as key regulators of immune system development and function. Therefore, it is important to determine miRNA dysregulation and its pathogenic contribution in autoimmune diseases, an aspect not adequately addressed thus far.

**Methodology/Principal Findings:**

In this study, we profiled miRNA expressions in splenic lymphocytes from three murine lupus models (MRL-lpr, B6-lpr and NZB/W_F1_) with different genetic background by miRNA microarray assays and Real-time RT-PCR. Despite the genetic differences among these three lupus stains, a common set of dysregulated miRNAs (miR-182-96-183 cluster, miR-31, and miR-155) was identified in splenocytes when compared with age-matched control mice. The association of these miRNAs with the disease was highlighted by our observation that this miRNA expression pattern was evident in NZB/W mice only at an age when lupus disease is manifested. Further, we have shown that the miRNA dysregulation in MRL-lpr mice was not simply due to the activation of splenocytes. By Real-time RT-PCR, we confirmed that these miRNAs were upregulated in both purified splenic B and T cells from MRL-lpr mice. miR-127 and miR-379, which were greatly upregulated in splenocytes from lpr mice, were moderately increased in diseased NZB/W mice. In addition, Real-time RT-PCR revealed that miR-146a, miR-101a, and miR-17-92 were also markedly upregulated in splenic T, but not B cells from MRL-lpr mice.

**Conclusions/Significance:**

The identification of common lupus disease-associated miRNAs now forms the basis for the further investigation of the pathogenic contribution of these miRNAs in autoimmune lupus, which will advance our knowledge of the role of miRNAs in autoimmunity. Given that miRNAs are conserved, with regard to both evolution and function, our observation of a common lupus disease-associated miRNA expression pattern in murine lupus models is likely to have significant pathogenic, diagnostic, and/or therapeutic implications in human lupus.

## Introduction

Systemic lupus erythematosus (SLE) is a chronic, complex, and debilitating systemic autoimmune disease. Patients often face a lifetime of illness with no effective cures and cope with high medical care costs. In past decades, the researchers have extensively focused on the identification of the gene defects in SLE. While there has been significant progress in the understanding of SLE genetics, which includes mapping of multiple SLE susceptibility loci/genes in the human and murine genome, to date no single or combination of structural gene defect has been identified as a principal pathogenic factor in inducing SLE [1], [2], [3]. Although genetic factors are important in SLE susceptibility, attention has now been shifted to the contribution of the epigenetic regulatory defects including abnormal DNA methylation, histone modification, and more recently miRNA regulation to lupus pathogenesis [2].

miRNAs are small (about 22 nucleotides), non-coding RNAs that regulate gene expression at the post-transcriptional level. miRNAs usually bind to the 3′ untranslated region (3′UTR) of target mRNAs through partial sequence homology, which result in either translation inhibition or degradation of target message RNAs (mRNAs). Computational analyses indicate that miRNAs constitute about 3% of the human genome and regulate at least 30% of human mRNAs. Therefore, it is not surprising that miRNAs have been shown to play essential regulatory roles in a variety of biological processes including embryogenesis, development, cell proliferation and apoptosis, and signaling transduction [4], [5], [6]. Importantly, aberrant miRNA expression patterns have been evident in various pathological conditions, which suggest the pathogenic roles of miRNA in different human diseases [5], [7]. A flurry of recent reports has documented the critical roles of miRNAs in regulating immune cell development, fine-tuning immune responses, and maintaining immune homeostasis [8], [9], [10], [11]. The involvement of miRNA in immune tolerance control and autoimmunity has also been suggested by recent studies in which autoimmunity was induced in mice when miRNA synthesis was selectively disrupted in regulatory T cells (Treg) or when miR-17-92 was over expressed in lymphocytes [12], [13], [14].

Spontaneous genetically lupus-prone murine models including NZBW_F1_/J (NZB/W), MRL/MpJ-Fas^lpr^/J (MRL-lpr), and congenic lupus strain B6.MRL-Fas^lpr^/J (B6-lpr) have been extensively used in numerous published reports and have provided valuable insight into lupus pathogenesis. These three strains share common characteristics with regards to the induction of autoantibodies against dsDNA and other nuclear components, but display different disease severity, autoantibody profile, and clinical manifestation, which are caused by the differences in the lupus-related genetic susceptibility loci and/or the background genome of these strains [1], [3], [15]. For example, although B6-lpr mice produce serum rheumatoid factor and anti-DNA antibodies, they do not develop obvious glomerulonephritis that is often noticed in MRL-lpr mice. This observation indicated that *Fas* gene mutation (lpr) alone is not sufficient for the development of glomerulonephritis. The interaction between lpr gene and MRL background genome is required for the development of renal disease in MRL-lpr mice [15], [16]. Intriguingly, in the present study, we identify a miRNA expression pattern in splenic lymphocytes that is common to all three lupus strains with different genetic background, and more importantly we demonstrate that this miRNA expression pattern is associated with lupus disease development.

## Results

### Identification of a common lupus-associated miRNA expression pattern in splenic lymphocytes from genetically lupus-prone mice

**Figure 1:**
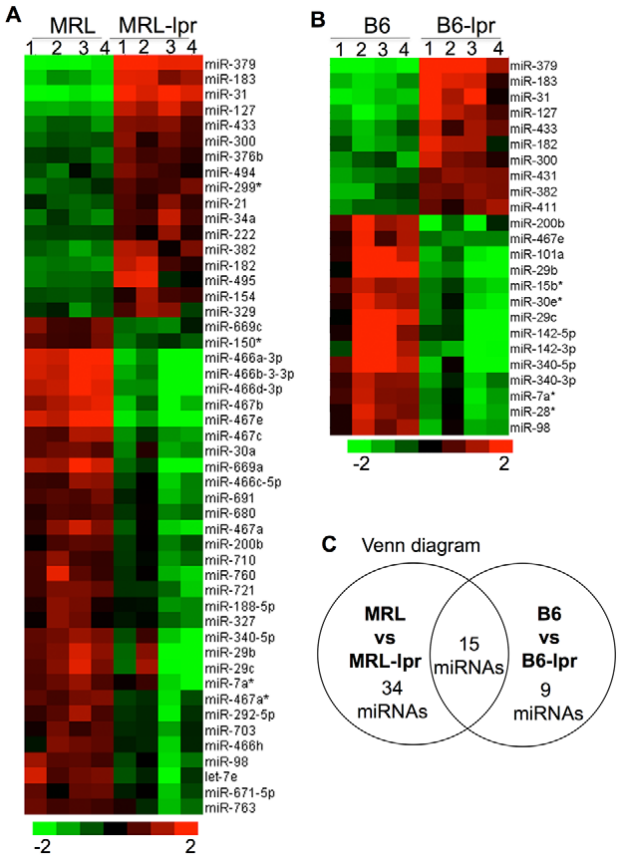
miRNA microarray data analysis. (A, B) Heat map representing the relative miRNA expression levels in splenocytes from MRL and MRL-lpr (A), and from B6 and B6-lpr mice at 3–4 months of age (B) (n = 4 each group). The miRNAs that demonstrated at least 3-fold changes with *p*<0.1, or 2-fold with *p*<0.05 were selected for hierarchical cluster analysis to generate the heat map. The color bar depicts the color contrast level of the heat map. Red and green indicate high and low expression levels, respectively. (C) Venn diagram presentation of microarray data.

To identify a lupus disease-associated miRNA expression pattern in splenocytes, we performed miRNA microarray assays to compare the miRNA expression profiles in splenocytes from three different strains of genetically lupus-prone mice including MRL-lpr, B6-lpr, and NZB/W mice and their respective control mice. To maintain uniformity of age, all of the mice were euthanized at 3-4 months of age. As indicated in figure 1, 49 miRNAs were differentially expressed in MRL-lpr mice when compared to MRL mice (Fig. 1A); and 24 miRNAs were differentially expressed in B6-lpr mice when compared to B6 mice (Fig. 1B). Among these dysregulated miRNAs, we noted that 15 miRNAs were common to both lpr strains (Fig. 1C and Table 1). The permutation test revealed that the possibility of randomly having an overlap of more than two miRNAs between dysregulated miRNAs in MRL-lpr and B6-lpr mice is <0.001. This indicated that the overlap of 15 dysregulated miRNAs observed in two lpr strains is significantly above the overlap by random chance (*p*<0.001). Interestingly, microarray analysis of NZB/W and NZW at 3–4 months of age, an age when overt lupus disease is not evident in NZB/W mice, revealed that only one miRNA, miR-148a, was significantly upregulated in NZB/W mice (data not shown).

**Table 1:** Microarray analysis revealed differentially expressed miRNAs in both MRL-lpr and B6-lpr mice.

		MRL-lpr/MRL		B6-lpr/B6	
	miRNA ID	*p* value	fold	*p* value	fold
**Upregulated**	mmu-miR-127	2.57E-06	6.87	1.86E-03	8.34
	mmu-miR-182	9.86E-04	4.08	6.06E-02	4.35
	mmu-miR-183	5.65E-06	7.57	5.84E-04	7.71
	mmu-miR-300	2.29E-03	2.58	5.64E-02	3.64
	mmu-miR-31	2.82E-07	16.22	1.87E-03	9.90
	mmu-miR-379	1.02E-07	16.80	7.34E-06	24.37
	mmu-miR-382	1.10E-03	3.66	1.90E-02	3.75
	mmu-miR-433	1.68E-04	3.48	1.06E-02	4.29
**Downregulated**	mmu-miR-200b	2.76E-02	-2.00	1.18E-02	-5.89
	mmu-miR-29b	2.76E-02	-4.76	3.91E-03	-18.28
	mmu-miR-29c	4.62E-02	-4.59	2.50E-02	-14.91
	mmu-miR-340-5p	1.22E-02	-3.73	7.29E-03	-9.96
	mmu-miR-467e	2.57E-06	-13.83	2.71E-02	-4.12
	mmu-miR-7a*	4.62E-02	-3.01	4.03E-02	-4.13
	mmu-miR-98	7.37E-03	-3.01	9.78E-02	-3.91

**Figure 2:**
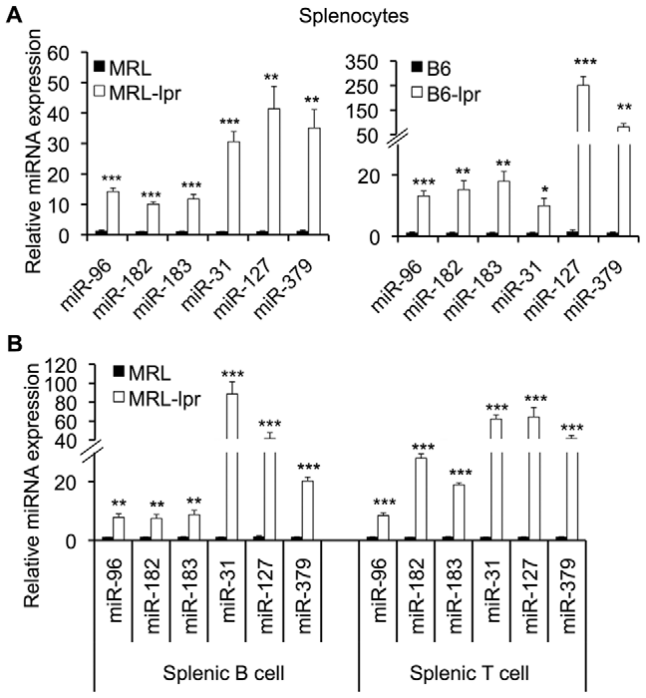
Real-time RT-PCR validation of dysregulated miRNA expression. (A) The graphs show miRNAs that were significantly upregulated in splenocytes from MRL-lpr and B6-lpr mice when compared to control MRL and B6 mice, respectively. (B) The graph shows miRNAs that are upregulated in both purified splenic B and T cells from MRL-lpr when compared to MRL mice. The graphs represent the means ± SEMs (n = 4 each). Student *t*-tests were performed. *, **, and *** indicate *p*<0.05, *p*<0.01, and *p*<0.001, respectively.

By Real-time RT-PCR analysis, we confirmed that the expression levels of miR-182, miR-183, miR-31, miR-127, and miR-379 were highly upregulated in splenocytes from both of MRL-lpr and B6-lpr mice when compared to control MRL and B6 mice, respectively (Fig. 2 A). A report has shown that miR-96 is clustered with miR-182 and miR-183 in mouse chromosome 6 and is likely generated from the same transcript [17]. Although microarray assay did not identify the change of miR-96 expression level in lpr mice, Real-time RT-PCR analysis clearly showed that miR-96 was markedly upregulated similar to two other members (miR-182 and miR-183) in the cluster (Fig. 2A).

Given the cell-specific expression of miRNA and divergent roles of a specific miRNA in different cell types, it is important to determine the expression of miRNA in purified subsets of splenic lymphoid cells. Therefore, we also analyzed the expression of miRNAs, which were altered in whole splenocytes, in purified B cells and T cells from MRL-lpr and control MRL mice using Real time RT-PCR. Consistent with the data observed in whole splenocytes, the expression of miR-182-96-183, miR-31, miR-127 and miR-379 was also significantly increased in purified splenic B cells and T cells from MRL-lpr mice when compared to MRL mice (Fig. 2B).

### Selected miRNAs were differentially altered in splenic B and T lymphocytes from lupus mice

**Figure 3:**
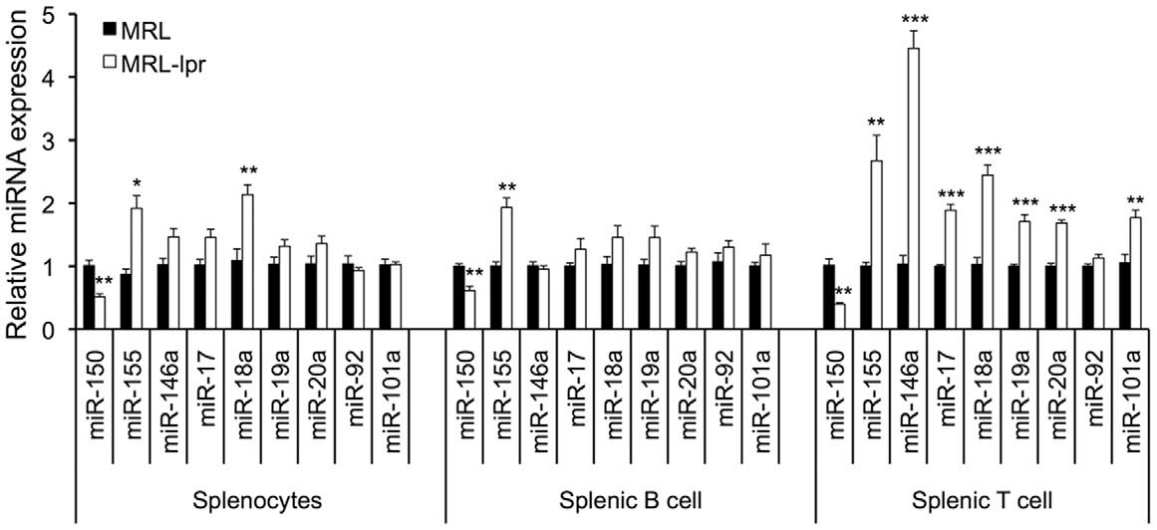
Real-time RT-PCR analysis of selected miRNAs. The expression levels of selected miRNAs were altered specifically in purified splenic T cells, but not B cells, from MRL-lpr when compared to MRL mice. The graphs represent the means ± SEMs (n = 4 each). Student *t*-tests were performed. *, **, and *** indicate *p*<0.05, *p*<0.01, and *p*<0.001, respectively.

A recent report revealed that miR-146a was decreased in peripheral blood leukocytes from human lupus patients, which contributed to the elevation of the type I interferon (IFN) pathway in human lupus [18]. Other studies have also revealed that miR-150, miR-155, miR-17-92, and miR-101a play roles in the regulation of antibody responses, germinal center responses, inflammatory responses, and/or autoimmunity [7], [13], [19], [20], [21]. Considering that miRNA microarray analysis is not as sensitive as Real-time RT-PCR to detect the alteration of the above-mentioned miRNAs in lupus mice, we performed Real-time RT-PCR to analyze the expression of above miRNAs in splenic lymphocytes. We found that miR-155 and miR-150 were significantly altered in whole splenocytes, as well as in splenic B and T cells from MRL-lpr mice when compared to MRL mice (Fig. 3). The upregulation of miR-155 was also observed in splenocytes from B6-lpr mice (data not shown). Impressively, we found that although several members of the miR-17-92 cluster (miR-17, miR-18a, miR-19a, miR-20a), miR-146a, and miR-101a were not changed in purified splenic B cells, they were significantly upregulated in splenic T cells (Fig. 3). However, miR-92, another member of the miR-17-92 cluster was not changed in either splenic B or T cells. Overall, our data revealed that the expression changes in lupus-associated miRNAs such as miR-182-96-183, miR-31, miR-127, miR-379, miR-155, and miR-150 that were observed in splenocytes were also evident in purified splenic B and T cells. However, some miRNAs including miR-17-92 cluster members, miR-146a and miR-101a were selectively dysregulated in splenic T cells, but not splenic B cells, suggesting an exclusive role of these miRNAs in lupus T cells.

### The dysregulation of miRNA expression in splenocytes is associated with lupus disease development

**Figure 4:**
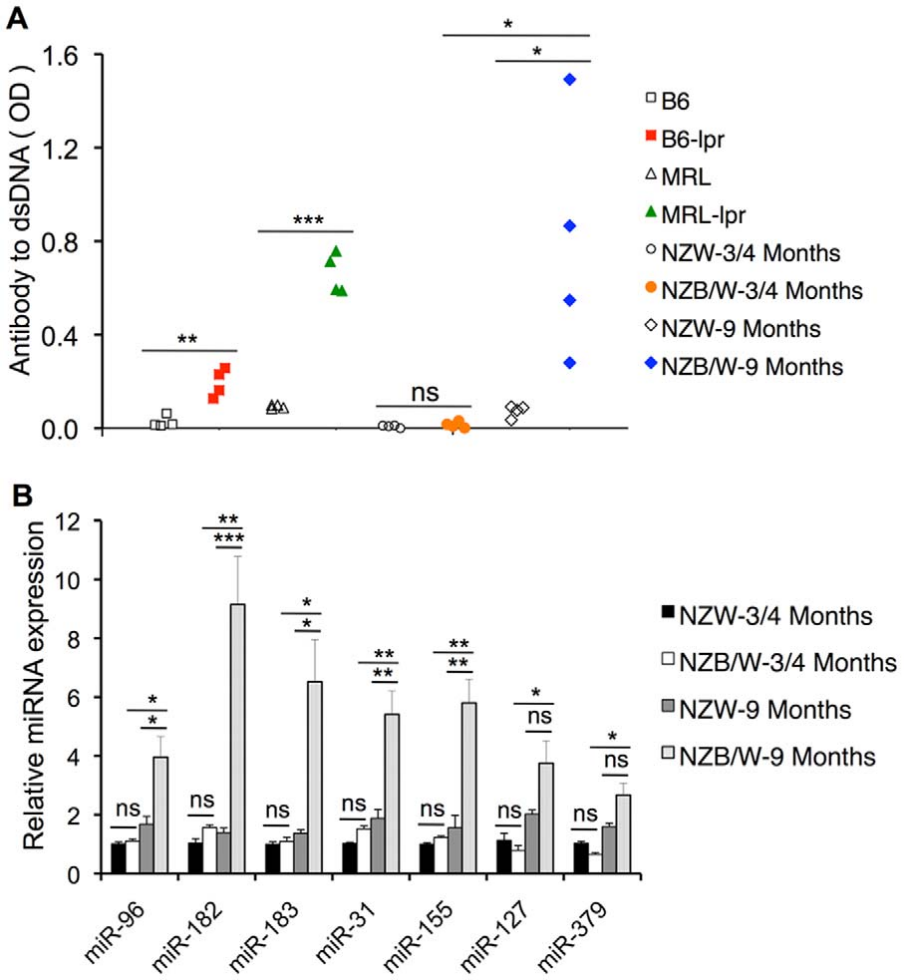
The alteration of miRNAs in splenocytes was related to lupus disease development. (A) The graph shows the serum anti-dsDNA autoantibody levels in different strains of lupus mice and their respective control mice (n = 4 each group). Student *t*-tests were performed. (B) Real-time RT-PCR analysis of miRNA expression in splenocytes from NZB/W and NZW mice at 3–4 and 9 months of age, respectively. The graph shows the means ± SEMs (n = 4 each). One-way ANOVA and the Tukey-Kramer multiple comparisons tests were performed. For both graphs, “ns” denotes non significant; *, **, and *** indicate *p*<0.05, *p*<0.01, and *p*<0.001, respectively.

As mentioned earlier, the miRNA changes observed in two lpr strains were not evident in 3–4 month old NZB/W mice when compared to age-matched NZW mice. It is important to note that at 3–4 months of age, lpr mice with *Fas* gene defects already manifest lupus parameters such as the generation of anti-dsDNA autoantibodies, an important hallmark of lupus disease. As shown in figure 4A, the serum anti-dsDNA levels were significantly increased in MRL-lpr mice and B6-lpr mice when compared to control MRL and B6 mice, respectively. Nevertheless, these lpr mice only had moderate mesangial hyperplasia/inflammation in the glomerulus and did not produce proteinuria (data not shown). There was no obvious anti-dsDNA production in either NZB/W or NZW mice at 3–4 months of age (Fig. 4A). Therefore, we utilized 9-month old NZB/W female mice, which had confirmed high serum levels of anti-dsDNA autoantibodies (Fig. 4A) and severe glomerulonephritis and proteinuria (data not shown), to analyze whether miRNA changes observed in lpr lupus mice are evident in 9-month old NZB/W mice. To avoid the potential effect of age on miRNA expression, we also included 9-month old NZW female mice as controls. Impressively, the expression levels of miR-182-96-183, miR-31, and miR-155 were markedly upregulated in 9-month old NZB/W mice when compared to either 9-month old NZW or 3-4-month old NZB/W mice (Fig. 4B). miR-127 and miR-379 were also significantly upregulated in 9-month old NZB/W mice when compared to 3-4-month old NZB/W. However, the upregulation of miR-127 and miR-379 in 9-month old NZB/W is not significant when compared to 9-month old NZW mice (Fig. 4B). These data strongly indicated that the dysregulation of above miRNAs in splenic lymphocytes is common in three lupus strains rather than specific to lpr mice.

**Figure 5:**
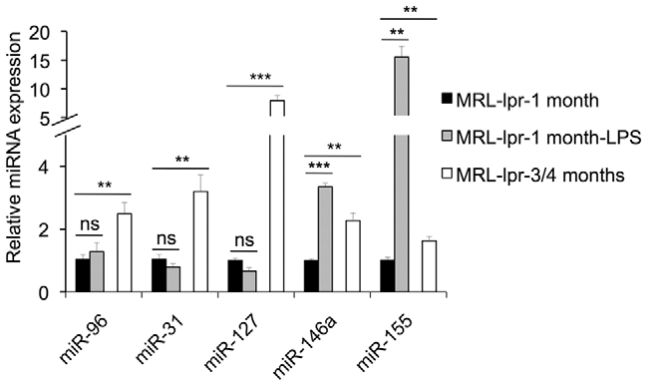
The dysregulation of lupus-associated miRNAs in lpr mice is not simply due to the activation of splenocytes. The expression levels of selected lupus-associated miRNAs (miR-96, miR-31, miR-127, miR-146a, and miR-155) in freshly-isolated (MRL-lpr-1 month), 24 hrs of LPS activated (MRL-lpr-1 month-LPS) splenocytes from 1-month old MRL-lpr mice, and freshly isolated splenocytes from 3-4 month old MRL-lpr mice (MRL-lpr-3-4 months) were analyzed by Real-time RT-PCR. The graph shows means ± SEM (n = 4 each). ns, non significant, **, *p*<0.01, and ***, *p*<0.001, MRL-lpr-1 month *vs.* MRL-lpr-1 month-LPS (paired *t* test), MRL-lpr-1 month *vs.* MRL-lpr-3/4 months (two tail *t* test).

To further determine whether the changes of lupus-associated miRNAs are simply a result of activation of lymphocytes in lupus mice or are associated with the lupus disease development, we analyzed the expression of selected lupus-associated miRNAs in freshly-isolated and lipopolysaccharide (LPS)-activated splenocytes from younger MRL-lpr mice (approx. 1 month-old), an age when these mice do not develop significant levels of serum anti-dsDNA autoantibodies. We also included freshly-isolated splenocytes from 3–4 months old MRL-lpr mice, which manifest lupus as positive controls. As shown in the figure 5, the expression levels of selected lupus-associated miRNAs including miR-96, miR-31, miR-127, miR-146a and miR-155 were significantly upregulated in freshly-isolated splenocytes from 3-4-month old MRL-lpr mice when compared to 1-month old MRL-lpr mice. This data further suggested that the upregulation of these miRNAs in lupus mice was associated with the lupus development. LPS stimulation significantly increased the expression of miR-146a and miR-155, which was consistent with the previously published reports in which miR-146a and miR-155 were shown to be induced by LPS [22], [23]. However, LPS activation did not induce miR-96, miR-31 and miR-127 expression changes in splenocytes. Taken together, our data strongly indicated that the common changes of miRNAs in lpr and NZB/W mice are associated with lupus disease development rather than a simply result of the activation of lymphocytes in lupus mice.

## Discussion

It is becoming apparent that miRNA plays an important role in immune homeostasis and also in the development or prevention of autoimmunity. Dysregulated miRNA expression has been evident in several human autoimmune diseases including multiple sclerosis (MS), rheumatoid arthritis (RA), and SLE [7], [18], [24], [25], [26], [27], [28]. However, the study of the role of miRNA in autoimmunity is still in the beginning stage. The precise role of dysregulated miRNAs as autoimmune disease biomarkers and the function of these miRNAs in the autoimmune disease process need to be determined.

Thus far, several reports have revealed abnormal miRNA expression patterns in peripheral blood mononuclear cell (PBMC) and renal biopsy samples from human lupus patients, respectively [18], [24], [25], [29], [30]. Intriguingly, it seems that the dysregulated miRNAs in lupus patients that were determined in one study are not well reproduced in other studies. For example, the decrease of miR-146a in PBMC from lupus patient was observed in the study reported by Tang et al. [18], but not in other published lupus studies [24], [25], [29]. The discrepancy of the data from different human lupus miRNA studies may be due to the difference in the type of samples, sensitivity of detection methods, and the diversity in the medical history, disease severity and race of the lupus patients. Considering that genetic factors contribute significantly to lupus etiology and may affect miRNA expression, it will be important to determine a miRNA expression pattern that is associated with lupus disease, but is independent of the genetic background of patients or animal models.

**Figure 6:**
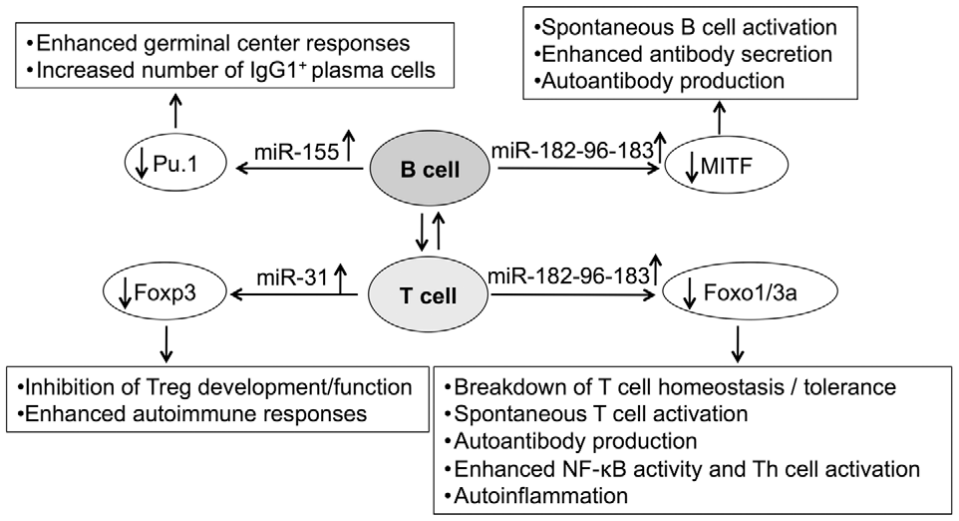
Outline of the potential contribution of the common lupus disease-associated miRNAs to B and T lymphocyte-mediated lupus pathogenesis.

In this study, we reported a common lupus disease-associated miRNA expression pattern in three murine lupus models with different genetic backgrounds. The reported targets and immune regulatory functions of the lupus disease-associated miRNAs suggest the potential contribution of these miRNAs to immune tolerance breakdown and altered B and T lymphocytes function in lupus mice (Fig. 6). For example, miR-155 has been shown to regulate germinal center cell responses and generation of high-affinity antibodies by targeting Pu.1, a ETS family transcription factor playing critical role in the development of lymphoid cells [21]. While overexpression of miR-155 led to the enhanced germinal center responses and antibody production, depletion of miR-155 inhibited germinal center responses and reduced the number of memory B cells and IgG class switched plasma cells [20], [21]. Our findings of the up-regulation miR-155 in splenic B cells may have relevance with the reported abnormal B cell activation, enhanced germinal center responses and antibody production in human and murine lupus [31]. The upregulation of miR-31 in T cells may correlate with the deficiency of Treg cell development/function in lupus since miR-31 targets Foxp3, a critical transcription factor for Treg cell development and function [32].

Of particular interest here, we found that miR-182-96-183 cluster is remarkably upregulated in splenic lymphocytes (over 8 folds in lpr mice) from lupus-prone mice. Previous study has shown that miR-182-96-183 cluster was primarily expressed in the retina, and rarely detected in the spleen and other tissues from normal B6 mice [17]. Our findings are the first to show that this cluster is selectively increased in splenocytes from lupus-prone mice, which may have a potential immune regulatory role in autoimmune mice. Of particular relevance, the published reports have shown that miR-182 and miR-96 target transcription factors Foxo1, Foxo3a, and microphthalmia-associated transcription factor (MITF), which play critical roles in controlling T and B lymphocyte homeostasis and tolerance, respectively [33], [34], [35], [36], [37]. T cell-specific deletion of Foxo1 gene in mice led to spontaneous T cell activation, higher percentage of IFNγ, IL-17 secreting CD4^+^ T cells, anti-dsDNA autoantibody production, and tissue inflammation [34]. Inactivation of Foxo3a in mice led to NF-κB activation, hyperactivation of helper T cell with increased Th1/Th2 cytokine production and autoimmune inflammation in salivary gland, lung and kidney [33]. Moreover, inactivation of MITF in mice induced spontaneous B cell activation and autoantibody production by suppressing interferon regulatory transcription factor (IRF)-4, which suggested a role of MITF in maintaining B cell immune tolerance and prevention of autoimmunity [35]. Therefore, the upregulation of miR-96-182-83 in lupus lymphocytes may cause the decrease of Foxo1/3a and/or MITF, which in turn leads to the hyperactivation of B and T lymphocytes, immune tolerance breakdown and development of autoimmunity. Consistent with this, there is decreased Foxo1 transcript in human SLE and inhibited Foxo3a expression and activity in MRL-lpr mice ([33], [38] and unpublished lab data).

In addition, we found that miR-101a, miR-146a and miR-17-92 cluster (except miR-92) were upregulated in splenic T cells from MRL-lpr mice. Further studies will be performed to determine whether these miRNAs are also altered in splenic T cells from other murine lupus models. The upregulation of miR-101a is consistent with the previous finding that miR-101a is upregulated in CD4^+^ T cells from *sanroque* mice, which develop lupus-like autoimmune syndrome as a result of loss of Roquin mediated-repression of the inducible T-cell co-stimulator (ICOS)[39]. Increased miR-17-92 in T cells may lead to T cell tolerance breakdown in MRL-lpr mice since over expression of miR-17-92 miRNA cluster in lymphocytes promoted lymphocytes proliferation and induced autoimmunity by targeting Bim and Pten, two molecules that play critical role in immune tolerance mechanism [13]. Our finding that miR-146a was significantly upregulated in splenic T cells from MRL-lpr mice is consistent with recent reports that have shown miR-146a was increased in splenic CD4^+^ T cell from MRL-lpr mice [40].

In this study, we are the first to report a common dysregulated miRNA expression pattern in splenocytes from three murine lupus models with different genetic background. Although we have shown that the dysregulated miRNAs were associated with the production of anti-dsDNA autoantibodies and the development of the lupus, it still needs to be determined whether the dysregulated expression of these miRNAs are exclusive to lupus (i.e. lupus-specific). It is plausible that some of these miRNAs may also be dysregulated in other autoimmune diseases. It is noteworthy that MRL-lpr and NZB/W mice, in addition to serving as animal models of human lupus, have also been extensively used as models of other human autoimmune diseases such as RA or Sjögren's syndrome [41], [42]. Therefore, the selected lupus-associated miRNAs that were determined in these three lupus models may also play a role in the pathogenesis of RA and/or Sjögren's syndrome. Indeed, miR-146a and miR-155 have been shown upregulated in PBMC and CD4^+^ T cells from human patients with RA and contributed to RA pathogenesis [43], [44].

The identification of a common lupus disease-associated miRNA expression pattern in splenic lymphocytes from different lupus-prone mice now forms the basis of the further investigation to mechanistically understand the pathogenic contribution of these miRNAs in autoimmune lupus. In figure 6, we outlined the potential contribution of the common lupus-associated miRNAs to B and T lymphocyte-mediated lupus pathogenesis based on the documented role of these miRNAs or their target genes in immune system. We are currently conducting exhaustive in depth studies to test these suppositions and to determine the direct contribution of these miRNAs in lupus pathogenesis. Given that miRNAs are evolutional conserved and that the murine lupus models used in this study resemble the major characteristics of human lupus, we believe that these studies will potentially open a new approach for lupus diagnosis and develop novel strategy for treating of lupus disease by altering lupus-specific miRNAs in lymphocytes.

## Materials and Methods

### Ethics

This study has been approved by the Virginia Tech Institutional Animal Care and Use Committee under Protocol ID #08-124-CVM.

### Mice

Genetically lupus-prone female mice MRL-lpr, NZB/W, B6-lpr, and their control mice MRL/MpJ (MRL), NZW/LacJ (NZW), and C57BL/6J (B6) mice were purchased from The Jackson Laboratory, ME, USA. All mice were housed in the animal facility at the CMMID, Virginia-Maryland Regional College of Veterinary Medicine, Virginia Tech. At 3–4 months of age (a time point when lpr mice develop lupus), MRL-lpr, B6-lpr, NZB/W and their control mice were euthanized and spleen tissues were collected to isolate splenocytes. Since NZB/W mice do not develop fulminant lupus at 3–4 months of age, additional groups of NZB/W and control NZW mice were euthanized at 9 months of age, when NZB/W develop classic lupus symptoms. In addition, a group of MRL-lpr mice at 1 month of age, an age when MRL-lpr mice do not develop significant level of anti-dsDNA autoantibodies, were also euthanized for the experiment.

### Splenocyte preparation, splenic B and T cell purification

Whole splenocytes were isolated using standard procedures described in detail previously [45], [46]. Splenic T cells and B cells were purified from freshly-isolated splenocytes sequentially using CD90.2 and CD19 microbeads (Miltenyi Biotec), respectively by positive selection per the manufacturer's instruction. The purity of isolated T cells and B cells was confirmed by flow cytometry after staining the isolated cells with FITC-conjugated CD90.2 (Thy1.2) and CD19 antibodies, respectively. For LPS stimulation, the splenocytes from 1-month old MRL-lpr mice were adjusted to 5×10^6^ cells/ml, plated in 24-well plate, and then activated for 24 hrs by adding equal volumes of media containing LPS (Sigma, 1000 ng/ml) to seeded cells (final concentration at 500 ng/ml).

### miRNA isolation and miRNA Microarray assay

Total RNA, containing miRNA, was isolated from whole splenocytes and purified splenic B and T cells using mirVana miRNA isolation kits (Ambion). The total RNA samples from whole splenocytes were sent to LC Sciences (http://www.lcsciences.com/) for the microarray assay as we described recently [47]. A μParaflo microfluidic chip, which included 617 unique, mature, mouse miRNA, based on the Sanger miRBase Release 12.0, was used for assay. The methods used for microarray data processing have been described in detail previously [47]. The miRNAs that demonstrated either at least 3-fold changes with *p*<0.1 or at least 2-fold changes with *p*<0.05 were selected for generation of the heat map and Venn diagram. The permutation test was performed to determine that the overlap of the dysregulated miRNAs observed in MRL-lpr and B6-lpr is a significant event rather than a random event. Briefly, out of 617 miRNAs on the chip, 400 miRNAs and 347 miRNAs are detectable in the samples of dataset MRL *vs.* MRL-lpr and dataset B6 vs. B6-lpr, respectively. Among them, 49 out of 400 miRNAs were differentially expressed in MRL-lpr mice when compared to MRL mice, and 24 out of 347 miRNAs were differentially expressed in B6-lpr mice when compared to B6 mice. The permutation test was performed by random sampling of 49 miRNAs from 400 miRNAs that were detectable in dataset of MRL *vs.* MRL-lpr and 24 miRNAs from 347 miRNAs that were detectable in dataset of B6 *vs.* B6-lpr. The procedure was repeated for 1000 times to determine the possibility of having an overlap of miRNAs in the randomly generated datasets.

All the microarray data are MIAME compliant and the raw data have been deposited in a MIAME compliant database (Gene Expression Omnibus (GEO)) under the accession number GSE22359.

### Real-time RT-PCR analysis of miRNA expression

As we previously described [47], the Taqman miRNA assay system (Applied Biosystems) was used to quantify miRNA expression. The relative expression level of miRNA was normalized to the endogenous small RNA control, snoRNA 202, and calculated using the 2^−ΔΔCt^ (Livak) method.

### Analysis of autoimmune parameters

Anti-dsDNA autoantibody ELISA was used to determine serum anti-dsDNA autoantibody levels as described previously [48]. Briefly, the Nunc MaxiSorp 96 well ELISA plates (Fisher Scientific) were coated with calf thymus dsDNA (Sigma, 100 µg/ml) overnight, blocked with PBS-1%BSA, incubated with series diluted serum samples, and then HRP conjugated goat-anti mouse IgG-gamma (Sigma). The signal was developed with TMB substrate (KPL, Inc). The plate was read at 380 nm in a VersaMax microplate reader (Molecular Devices). Proteinuria was measured by dipstick analysis using Chemistrip-2GP (Roche). The kidneys of mice were fixed in buffered formalin and subjected to histopathological assessment of glomerulonephritis by a pathologist (Dr. David Caudell) in a blind fashion as previously reported [49].

### Statistical Analysis

All values in the graphs are given as means ± SEM. To assess statistical significance, student *t*-tests or one-way ANOVA and the Tukey-Kramer multiple comparisons tests were performed using GraphPad InStat (version 3.0a for Macintosh).
